# Editorial: Neuroarthrology: Exploring anatomy, molecular biology, and the nervous system in osteoarthritis

**DOI:** 10.3389/fmed.2023.1136981

**Published:** 2023-01-24

**Authors:** Veronica Macchi, R. Shane Tubbs, Francisco Airton Castro Rocha

**Affiliations:** ^1^Institute of Human Anatomy, Department of Neuroscience, University of Padua, Padua, Italy; ^2^Departments of Neurosurgery, Neurology, and Structural and Cellular Biology, School of Medicine, Tulane University, New Orleans, LA, United States; ^3^Department of Internal Medicine, Faculdade de Medicina da Universidade Federal Do Ceará, Fortaleza, Brazil

**Keywords:** osteoarthritis, neuroarthrology, synovium, neuroanatomy, regenerative medicine

Osteoarthritis (OA) is a musculoskeletal disease that carries the highest social and economic burden to society, being among the major causes of years lost due to disability (1). Diagnosing OA is not a major issue in clinical practice. However, the lack of adequate biomarkers severely limits the development of novel therapies, thus correctly determining their prognosis (2). OA occurs due to a low-grade inflammatory process inside the joints, leading to their failure, and it has gained considerable attention in recent years (3). Thus, tackling the inflammatory process happening in the joint, which may be shared by comorbidities frequently associated with OA, such as obesity and cardiovascular disorders, may offer opportunities to develop disease-modifying OA drugs (4). Unfortunately, while we wait for strategies (pharmaceutical or not) to halt disease progression, patients with OA are experiencing pain and functional incapacity. Additionally, pain control in this disease is also far from ideal (5). Therefore, there is a need for developing safer and more effective alternatives for managing pain in patients with OA. Several articles on this Research Topic have attempted to address clinical aspects as well as the neuroanatomical and molecular pathways involved in pain mechanisms and structural damage to the joints affected by OA. Although understanding the pathogenesis of the disease is the most immediate perceived objective, we believe that new data will finally provide options to improve the treatment of a patient with OA.

Regenerative medicine is related to the mechanisms that attempt to either halt or repair damage to joint tissues in various diseases. Although bone cells and synoviocytes can divide the chondrocyte, being the one and only actor to produce articular cartilage, it has a very limited, if any, capacity to regenerate. Mesenchymal stem cells (MSCs) collected locally, e.g., inside the joint or transferred from other tissues after *in vitro* treatment, have been used as a strategy to regenerate cartilage (6). One of the drawbacks of this approach is that the MSCs are insufficient to determine the expression of SOX9 (SRY-type high mobility group box-9), a chondrogenic transcription factor (7). Zhang et al. discussed the possibility that the intra-articular administration of super-positively charged SOX9 could stimulate endogenous MSCs inside the joints, thereby helping to regenerate the damaged cartilage.

Metabolic changes linked to comorbidities associated with OA have long been implicated in the mechanisms driving joint damage in this disease (8). Cells in the adipose tissue are involved in this metabolic imbalance, also called metainflammation, which is linked to the low-grade inflammation that happens in OA, obesity, and metabolic syndrome (9, 10). However, it might be the fact that adipose tissue cells from different sources behave differently. Stocco et al. compared the non-inflamed infrapatellar fat pad (IFP) from healthy young and elderly individuals. They found morphological differences as well as changes in the type of collagen and the elastic fiber between both groups. These changes led them to speculate that alterations linked to the aging process in non-inflamed IFP could be secondary to persistent mechanical stimuli. The relevance of changes to the subchondral bone in OA pathogenesis is also established and varied. It may not only result from mechanical changes secondary to daily life activities damaging the ligaments, the tendons, and/or the muscles surrounding the joints but also due to the contribution of the bone marrow cells toward the release of inflammatory mediators in addition to driving osteophyte formation. Using an *in vitro* approach, Mukai et al. isolated bone marrow-derived monocytes/macrophages (BMM) that were then treated *in vitro* with an Lv peptide, a fragment from the V-Set and transmembrane domain containing four molecules (VSTM_4_), which has the immunosuppressive activity against T cells and the vascular endothelial growth factor (VEGF). The Lv peptide provoked a decrease in the release of cytokines by BMM following stimulation with bacterial lipopolysaccharide (LPS), leading to the proposal that the Lv peptide might display anti-inflammatory activity in the synovial cells.

Epidemiological aspects concerning the burden of OA were the subject of the article by Shamekh et al. providing data on populations living in the Middle East and North Africa. In addition to reinforcing the relevance of OA as a major cause of years lost due to disability, they reported an increase in the burden from 1990 through 2019, calling for a need for preventive measures aiming to mitigate this problem. Another epidemiological aspect was discussed by Chiu et al. who conducted a retrospective study on the long-term risk of age-related macular degeneration (AMD) in Taiwanese patients affected by OA. These authors found an increased risk for AMD in patients with OA, regardless of age and sex, which adds to the burden of comorbidities associated with this disease.

Non-pharmacological therapies represent a “must do” strategy in treating patients with OA. For example, Tai Chi practice is considered a core treatment for OA, being advocated in the osteoarthritis research international (OARSI) treatment guidelines as recommendations for all patients with OA. Shen et al. explored neurobiological mechanisms and mediators linked to the benefits of Tai Chi practice in individuals with knee OA. This pilot study showed that Tai Chi practice led to a significant improvement in pain and function in patients with OA, which was associated with improvement in neurobiological connectivity assessed by magnetic resonance imaging of the amygdala. There were also changes in the levels of lysophosphatidylcholines and lipid metabolites. Although based on a small group of patients, this study adds to the bulk of data showing that systemic changes including modifications happening in the central nervous system are linked to the effects of treatment interventions for patients with OA.

We hope that the interested reader will not only enjoy reading the articles but also be stimulated to produce data related to the mechanisms of pain experienced by a patient with OA.

## Author contributions

All authors listed have made a substantial, direct, and intellectual contribution to the work and approved it for publication.
